# Jak-Stat Signaling Induced by Interleukin-6 Family Cytokines in Hepatocellular Carcinoma

**DOI:** 10.3390/cancers11111704

**Published:** 2019-11-01

**Authors:** Juliane Lokau, Victor Schoeder, Johannes Haybaeck, Christoph Garbers

**Affiliations:** 1 Department of Pathology, Medical Faculty, Otto-von-Guericke-University Magdeburg, 39120 Magdeburg, Germany; 2 Diagnostic & Research Center for Molecular BioMedicine, Institute of Pathology, Medical University of Graz, 8036 Graz, Austria; 3 Department of Pathology, Neuropathology, and Molecular Pathology, Medical University of Innsbruck, 6020 Innsbruck, Austria

**Keywords:** HCC, Jak1, STAT3, IL-6

## Abstract

Hepatocellular carcinoma (HCC) is one of the most common malignant tumors worldwide. It can be caused by chronic liver cell injury with resulting sustained inflammation, e.g., triggered by infections with hepatitis viruses B (HBV) and C (HCV). Death of hepatocytes leads to the activation of compensatory mechanisms, which can ultimately result in liver fibrosis and cirrhosis. Another common feature is the infiltration of the liver with inflammatory cells, which secrete cytokines and chemokines that act directly on the hepatocytes. Among several secreted proteins, members of the interleukin-6 (IL-6) family of cytokines have emerged as important regulatory proteins that might constitute an attractive target for therapeutic intervention. The IL-6-type cytokines activate multiple intracellular signaling pathways, and especially the Jak/STAT cascade has been shown to be crucial for HCC development. In this review, we give an overview about HCC pathogenesis with respect to IL-6-type cytokines and the Jak/STAT pathway. We highlight the role of mutations in genes encoding several proteins involved in the cytokine/Jak/STAT axis and summarize current knowledge about IL-6 family cytokines in this context. We further discuss possible anti-cytokine therapies for HCC patients in comparison to already established therapies.

## 1. Introduction

Hepatocellular carcinoma (HCC) is one of the most frequent malignant tumors worldwide. It ranks first among all liver tumors, which, in total, are one of most common entities related to the number of deaths after lung, colorectum and stomach cancer [1]. The underlying causes are manifold, but are usually caused by sustained inflammation, chronic damage to the liver, e.g., alcohol abuse or ingestion of liver-toxic compounds, or infections with hepatitis viruses B (HBV) and C (HCV). During these processes, hepatocytic injury leads to the activation of compensatory mechanisms, which can ultimately result in liver fibrosis and cirrhosis. The livers of HCC patients are often infiltrated with different types of inflammatory cells, depending on the pathogenic agent. Importantly, nonalcoholic steatohepatitis (NASH) is a rising cause of HCC [2], and it is estimated that its role in HCC development will further increase [1].

The Jak/STAT signaling cascade is a central signaling hub that can be activated by a plethora of cytokines, growth factors and hormones [3]. It controls different cellular processes, including cell division, proliferation and cell fate decision. It consists of seven mammalian STAT family members that act as transcription factors and are activated by four different Janus kinases [4]. Besides its ability to convert extracellular stimuli to intracellular changes in the gene expression profile of the cell, several mutations in central proteins of the Jak/STAT pathway are known that constitutively activate the pathway without the need of a stimulus from outside of the cell [5].

In this review article, we summarize the etiology and mechanisms of hepatocarcinogenesis and describe the currently known functional consequences of Jak/STAT signaling in HCC. We focus on the interleukin-6 (IL-6) family of cytokines as the extracellular activator of the signaling cascade and report the contribution of activating mutations. Furthermore, we discuss possible strategies for targeting the Jak/STAT pathway or certain extracellular cytokines in HCC therapy.

## 2. Etiology and Development of Hepatocellular Carcinoma (HCC)

HCC is by far the most frequent of all primary liver malignancies, making up for at least 75% of all of them. It is the sixth most common cancer and the fourth most cancer-related cause of death worldwide [6]. In the vast majority of cases, the development of the various subtypes of HCC can be attributed to a defined cause [7]. About 80% of all HCC patients have an associated cirrhotic liver [8]. 

The underlying reason is a chronically incorporated or constantly active hepatotoxic agent [9], e.g., viral infections (mostly hepatitis viruses types B and C [10,11,12]) or chronic alcohol abuse. The latter and lifestyle-associated metabolic disorders are held responsible for the development of most liver cancers, especially in the so-called Northern Hemisphere [13]. However, non-alcoholic causes are also known, e.g., in the context of metabolic syndrome [14,15] like diabetes mellitus and dismal nutrition [13,16]. In addition, numerous exogenous substances can initiate hepatocellular carcinogenesis, like fungal toxins such as aflatoxin B1, which is especially important in tropical and subtropical regions [17]. Besides these rather common noxae, suffering from inherited malady, e.g., haemochromatosis or glycogen storage diseases, are rather seldom but relevant triggers for HCC formation [9].

**Figure 1:**
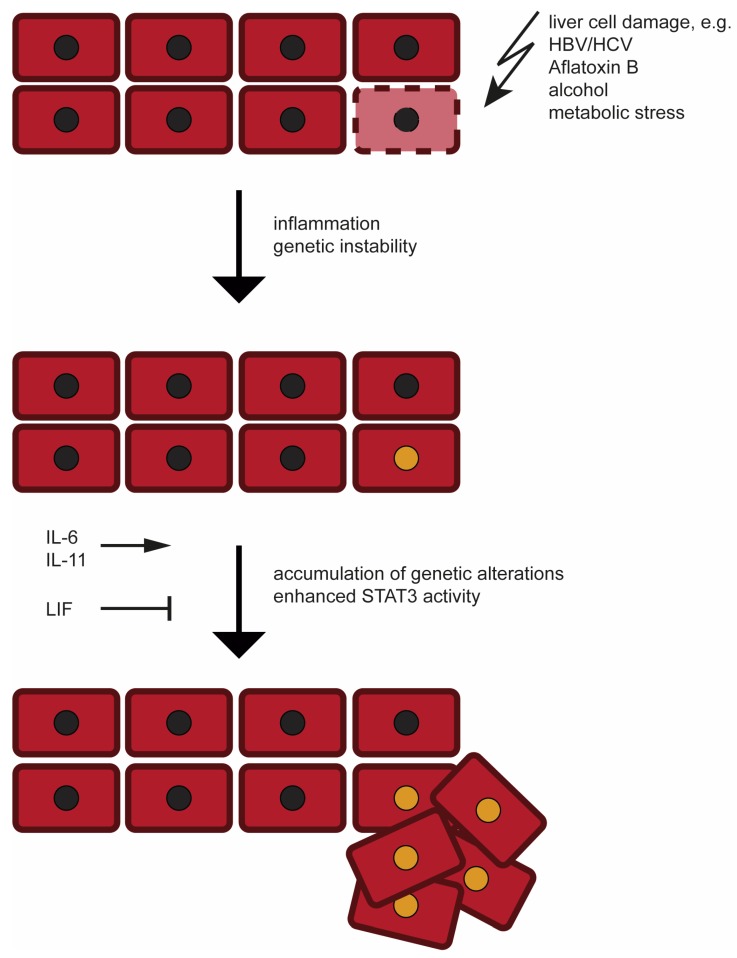
Schematic Depiction of HCC Development. Hepatocellular carcinogenesis is initiated by hepatocyte damage resulting in accumulation of genetic alterations and progression towards malignant liver cells. More details are given in the main text.

From the current state of knowledge, the development of HCC follows a cascade of increasing accumulation of genetic alterations [18,19]. Cirrhotic or non-cirrhotic parenchymal changes associated with chronic liver disease form the basis for the emergence of premalignant conditions leading to HCC [18,19] (Figure 1). Notwithstanding, especially in non-cirrhotic liver disease like chronic hepatitis B, the de novo-cancerogenesis is a well-known phenomenon [8]. In the gradual progression from a hepatocyte to the clonal proliferation of malignant liver cells, the first step is thought to be the dysplastic focus—a microscopic lesion with little atypia [20]. This alteration directly leads to the dysplastic nodule, a lesion that is frequently identifiable by radiological criteria and confirmed by histology [21]. Low- and high-grade dysplastic nodules are defined over the existence of so-called small cell changes on the one hand and large cell changes on the other hand [22]. These changes remain present in manifested HCC as well. The most striking, but not the only, criterion to differentiate these two forms of alterations in a dysplastic nodule is the cell size in comparison to the pre-existing hepatocyte [23]. Discrimination between low- and high-grade dysplasia is mostly based on the overall structural appearance and the size variation between individual cells within the particular dysplastic nodule [24].

The malignant potential of low-grade dysplastic nodules remains unclear. In contrast, high-grade dysplastic nodules periodically evolve into highly differentiated HCC [25]. These tumors are called small and early HCC [23]. If they remain undiscovered and untreated, they are believed to progress [23]. Subsequently, a larger, potentially lower differentiated and even higher malignant lesion will expand. HCC are defined by broadened liver cell cords and loss of the original reticulum network. This progress will often culminate in intra- and afterwards, extrahepatic metastasis. At that particular stage, the prognosis is devastating [26].

An important subtype of HCC is the steatohepatitic subtype, which is defined by the presence of intratumoral steatosis and inflammation. It accounts for up to 20% of all HCCs and is the archetypical liver cancer on the background of fatty liver disease, either alcohol-driven (arisen in Alcoholic Steatohepatitis, ASH) or not (Non-Alcoholic Steatohepatitis, NASH) [27,28,29]. A key molecular feature of steatohepatitic HCC is the activation of the IL-6/JAK/STAT signaling pathway [30].

HCC is an example of an extremely heterogenous cancer entity that displays a wide variety of genetic and molecular alterations [31], including mutations in members of the IL-6/Jak/STAT signaling cascade (see Section 5 for more details). Furthermore, telomere shortening, early-occurring activation of TERT promoter (as driver gene incident, [32,33,34]) and cell-cycle checkpoint regulator inactivation [35], e.g., of p21 [36,37], are some events of gradually increasing genetic changes [38]. Because the treatment of HCC is still challenging (see Section 6 for further details), establishing targeted therapeutics could lead to a certain advance in disease control.

## 3. The Jak/STAT Signaling Cascade

One important family that activates the Jak/STAT signaling cascade is the interleukin-6 (IL-6) family of cytokines [39]. This family consists of IL-6, IL-11, IL-31, cardiotrophin-like cytokine (CLC), ciliary neurotrophic factor (CNTF), cardiotrophin-1 (CT-1), oncostatin M (OSM), leukemia inhibitory factor (LIF) and IL-27, which is a heterodimeric cytokine that consists of the two subunits EBI3 and p28 [39,40]. The hallmark of this family is the usage of the signal-transducing β-receptor gp130. IL-6 and IL-11 activate a gp130 homodimer and need an additional unique non-signaling α-receptor (IL-6R and IL-11R) for complex formation [41]. CLC and CNTF bind first to the GPI-anchored CNTF receptor (CNTFR) before activating a heterodimer of gp130 and LIF receptor (LIFR). LIFR/gp130 are also activated by CT-1, OSM and LIF, but these three cytokines can engage their β-receptors directly and do not need an additional α-receptor. OSM can furthermore signal via gp130 in combination with the OSM receptor (OSMR), and IL-27 activates a heterodimer of gp130 and WSX-1. The only exception is IL-31, which signals through a heterodimer of OSMR and gp130-like protein (GPL) [42].

Binding of IL-6-family cytokines to their cognate receptors induces homo- or heterodimerization of the signal transducing receptor glycoprotein (gp)130. This dimerization induces the activation of different intracellular signaling cascades, including PI3K/Akt-, MAPK-, Yap-cascade, and, most prominently, the Jak/STAT cascade [39,43,44]. The Jak/STAT pathway is a common signaling cascade that is shared by various cytokines and consists of four Jaks and seven STATs, albeit not all of them are equally involved in signal transduction for a specific extracellular molecule. Jak/STAT signaling is essential in numerous physiological processes and consequently involved in different diseases [4].

**Figure 2:**
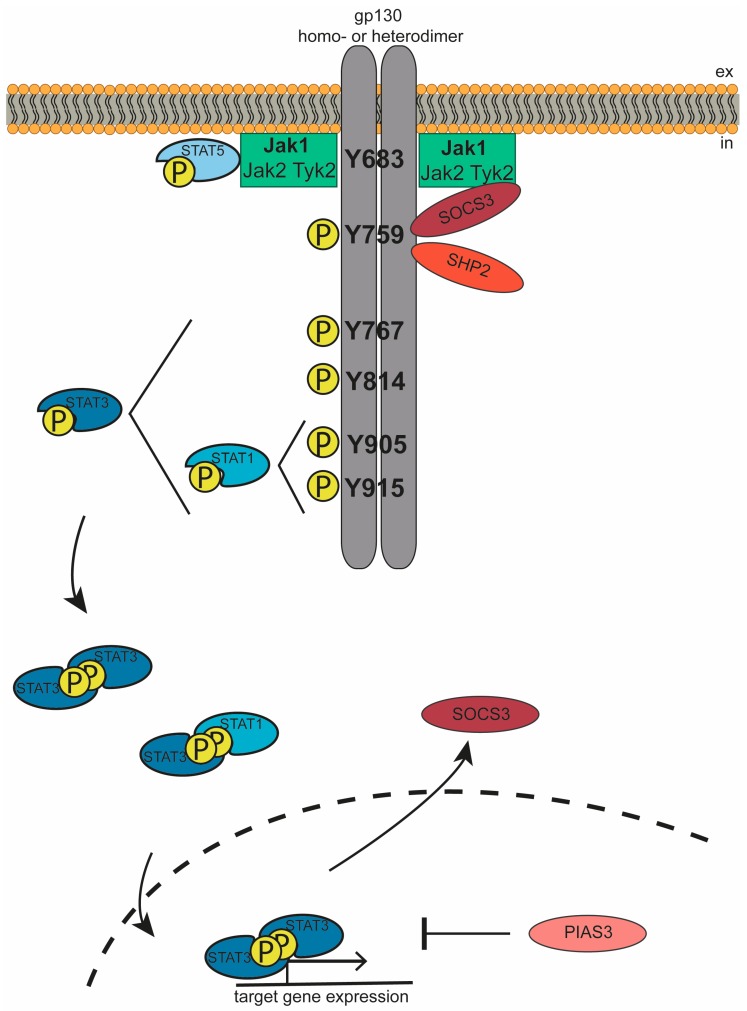
gp130-Mediated Jak/STAT Signaling. Upon cytokine binding, gp130 forms active homo-or heterodimers. This results in activation of intracellularly associated Janus kinases, which subsequently phosphorylate gp130 and STAT transcription factors, resulting in expression of target genes. Jak/STAT signaling is negatively regulated by SOCS3, PIAS3, and SHP2. More details are given in the main text.

Gp130 is intracellularly constitutively associated with three members of Jak family, namely Jak1, Jak2, and Tyk2 [45]. The association of Jaks is dependent on two membrane-proximal motifs within the gp130 intracellular region: Box1 is a proline-rich sequence, while Box2 is a rather hydrophobic region [46,47,48]. Loss of either of these motifs prevents ligand-induced phosphorylation of gp130 [46]. Of the associated kinases, only Jak1 appears to be essential for gp130 signaling since genetic ablation of Jak1 severely reduces signaling of IL-6 family cytokines, while lack of Jak2 or Tyk2 had no substantial impact [49,50,51,52]. The binding of cytokines to their receptors results in an altered intracellular architecture [53], which brings the Jak kinases into close proximity, resulting in their autophosphorylation and activation [45]. The activated kinases then phosphorylate distinct tyrosine residues in the intracellular part of gp130, which serve as docking sites for STATs [54,55]. Mutational analysis revealed that STAT3 interacts with the four membrane-distal tyrosine residues pTyr767, pTyr814, pTyr905, and pTyr915, while STAT1 only binds to pTyr905 and pTyr915 [54,55]. Upon binding of the transcription factors to the phosphorylated gp130, STAT1/3 are also phosphorylated by Jak kinases, and thus activated [56]. Furthermore, STAT5 is also activated upon gp130 activation, albeit to a lesser extent. In contrast to STAT1/3, however, STAT5 does not bind to the phosphorylated gp130 but rather, interacts directly with the Jak kinases [57]. STAT1 and STAT3 are phosphorylated at Tyr701 and Tyr705, respectively [58], while activated STAT5 is phosphorylated at Tyr694 [59]. Phosphorylation of STATs results in dissociation from the receptor and reorientation in active homo- or heterodimers, which regulate the transcription of target genes [56,60,61] (Figure 2).

One of the target genes is the suppressor of cytokine signaling 3 (SOCS3), the main negative regulator of gp130-mediated signaling [62,63]. SOCS3 binds to phosphorylated Tyr759 in gp130 [64,65] and thus inactivates signaling via two distinct mechanisms. Firstly, it directly inhibits Jak activity through binding of its kinase inhibitory region to the kinase domain of Jak1, Jak2, or Tyk2, which prevents further phosphorylation of STATs [66,67,68]. Further, through binding to gp130 and Jak, SOCS3 promotes the proteosomal degradation of these molecules [65,69,70]. The Tyr759 residue is also the binding interface for another negative regulator, namely Src-homology-2-domain-containing phosphatase 2 (SHP2) [54,64,71,72]. In contrast to SOCS3, SHP2 does not represent a feedback inhibition but is constitutively expressed and interacts with gp130 directly upon phosphorylation of the receptor, thus rather regulating intensity than length of STAT activity [47,73]. Of note, SHP2 does not only act as negative regulator for STATs, but is also phosphorylated upon gp130 activation and subsequently, activates the PI3K and MAPK signaling cascades [43]. Another negative regulator of gp130 signaling is protein inhibitor of activated STAT (PIAS), which binds to phosphorylated STATs, thus preventing binding of the transcription factors to DNA [74,75].

## 4. IL-6 Family Cytokines in HCC Development

The best-characterized member of the IL-6 family of cytokines in HCC is IL-6 itself, with a large body of data from human patients and corresponding experimental mouse models. Evidence for increased IL-6 levels in chronic liver diseases has already been described more than 25 years ago [76,77,78]. Most recently, a systematic review and meta-analysis confirmed elevated IL-6 serum levels in HCC patients compared to healthy controls, and showed that HCC patients had even higher IL-6 levels than patients with other chronic liver diseases like cirrhosis or hepatitis [79]. Furthermore, high IL-6 levels might be associated with poor prognosis of patients with advanced HCC [80]. Also, the fact that HCC occurs mainly in men has been causatively linked to IL-6, because estrogen inhibits the release of IL-6 from Kupffer cells, which reduces the risk of developing HCC in women [81]. An important cellular source of IL-6 appear to be the tumor-associated macrophages [82], although HCC cells themselves are also able to secrete IL-6 in a YAP-dependent manner [83].

Interestingly, the amount of sIL-6R in the circulation of HCC patients is also increased, suggesting a possible role for IL-6 trans-signaling in this process [84]. This is further supported by experiments in mice overexpressing the IL-6 trans-signaling inhibitor sgp130Fc, which show reduced tumor formation in the diethylnitrosamine/3,3′,5,5′-tetrachloro-1,4-bis(pyridyloxy)benzene model of HCC [85].

Besides IL-6, IL-11 has also been implicated in hepatocarcinogenesis. IL-11 expression has been demonstrated in the tumor tissue, and this expression pattern correlated with higher tumor node metastasis stage and was shown to be a prognostic factor for overall survival [86]. Intratumoral IL-11 expression was also identified as a prognostic factor for the development of bone metastasis [87,88]. Mechanistically, a long noncoding RNA activated by TGF-β (lncRNA-ATB) that is upregulated in HCC and is able to induce the epithelial–mesenchymal transition binds to the IL-11 mRNA and triggers STAT3 signaling through the autocrine induction of IL-11 production [89]. In addition, the long noncoding RNA Lnc34a is also overexpressed in HCC tissue and has a pro-metastatic function, at least partly by altering the TGF-β-induced IL-11 expression [90]. Recently, transmembrane p24 trafficking protein 3 (TMED3) has been shown to be upregulated in HCC and to be associated with poor prognosis for the patients. TMED3 appears to increase IL-11 expression and thereby promote HCC metastasis [91]. However, the involvement of TGF-β was not investigated and it is, therefore, unclear whether TMED3 and TGF-β independently promote IL-11 expression in HCC, or whether they are part of the same molecular pathway. Most recently, Wang and colleagues investigated possible mechanisms that underlie the risk of HCC recurrence after surgery [92]. They identify IL-11 as a key factor that increases after surgery and show that blocking of STAT3 signaling induced by IL-11 reduces proliferation of the tumor cells and, eventually, HCC [92].

The third well-characterized family member is LIF. LIF was shown to be highly expressed in HCC tumor samples [93]. Its expression was inversely associated with the microRNA miR-637, which was downregulated in HCC specimen and different HCC cell lines. Mechanistically, overexpression of mimics of miR-637 decreased STAT3 phosphorylation in HCC cell lines by suppressing the autocrine production of LIF [93]. LIFR expression has further been proposed as a biomarker for the differentiation of HCC from dysplastic nodules, showing that LIFR expression decreases as the tumor cells progress from low-grade dysplastic nodules to HCC [94]. Indeed, *LIFR* has been previously described as an HCC tumor suppressor gene that is silenced in HCC due to hypermethylation of the promotor of the *LIFR* gene [95]. This finding was later substantiated in a study showing that LIFR expression is not only reduced in HCC, but even further downregulated in HCC with metastasis [96].

Six- to seven-fold increased levels of OSM were detected in the serum of HCC patients compared to controls [97]. Similarly, serum levels of IL-27 were significantly increased in HCC patients compared to either healthy control patients or patients with other liver diseases like cirrhosis or hepatitis [98]. In contrast, a recent report showed decreased expression of EBI3, which is the subunit of IL-27 that resembles a soluble cytokine receptor, in HCC, which was associated with poor prognosis [99]. However, no functional role for these two cytokines has been established in HCC, and elevated serum levels might not be causatively linked to HCC. The role of the other members of the IL-6 family of cytokines in HCC is even less clear, with the exception of IL-31, which was reported to be largely absent in the serum of HCC patients [100].

Although the mechanisms of how IL-6-family cytokines induce HCC development are not entirely understood, the activation of the STAT3 signaling pathway, which is a hallmark of these cytokines, appears to be the underlying molecular cause.

## 5. Inborn Mutations in Proteins of the Jak/STAT Cascade in HCC Development

Various mutations and genetic alterations have been described to be involved in the onset and progression of HCC [101,102,103], and it has been reported that histological subtypes correlate with specific mutations [30]. According to Kan et al. and Nault et al., 45.5% of HCC patients display altered Jak/STAT signaling [33,104], although it must be noted that not all of these mutations are found in gp130-associated molecules.

Activating mutations in gp130 are found in 1–2% of HCC patients [105]. Mechanistically, the mutated receptors harbor small in-frame deletions in the cytokine binding site, which results in ligand-independent phosphorylation of STAT3 [105]. Notably, the mutated gp130 variants remain responsive towards inhibition by SOCS3 [106]. Furthermore, it could be shown that the phosphorylation of STAT3 was solely mediated by Jak1, but not Jak2 or Tyk2 [106]. Interestingly, these activating gp130 mutations are much more frequent in inflammatory hepatocellular adenomas (iHCA) than in HCC [105]. Furthermore, gp130 mutations accompanied by β-catenin mutations appear to promote malignant transformation of iHCAs [105]. Interestingly, in a mouse model for HCC, lack of gp130 in hepatocytes did not prevent initiation of HCC but attenuated cancer progression [107].

For the α-receptors of the IL-6 family, no mutations have been directly linked to HCC. However, several variants of these receptors are found in humans, which might affect HCC development in homozygous carriers. The frequent single-nucleotide polymorphism (SNP) rs2228145 in the IL-6R results in an exchange from Asp358 to Ala358 [108,109]. This IL-6R variant is much more prone towards proteolytic processing [110], resulting in an increase in circulating soluble IL-6R and a decrease in IL-6R amount on hepatocytes, thus an attenuated IL-6 response [108,109,111]. Furthermore, there are inactivating mutations in the IL-11R that results in loss of surface IL-11R and consequently in cells unresponsive towards IL-11 [112,113,114]. Carriers of these mutations would also not have any IL-11 response in hepatocytes.

Different point mutations in Jak1 have been described in HCC patients, which are predominantly located in the kinase and pseudokinase domains [104,115,116]. Notably, the majority of these mutations result in increased phosphorylation of Jak1 and STAT3 without stimulus. In contrast, IL-6-dependent Jak/STAT signaling was not altered with the Jak1 variants compared to wildtype Jak1 [104]. Most of the activating mutants are located at the pseudokinase/kinase domain interface and hypothesized to interfere with this interaction, thus preventing the inhibitory effect of the pseudokinase domain [104]. A similar mechanism has been proposed for the well-studied Jak2 mutant V617F [117]. Importantly, some of the Jak1 mutations that were identified in HCC patients showed no functional difference from wildtype Jak1 in vitro [104,116]. It has recently been proposed that the mutational status of Jak1 could be used to predict the efficacy of Jak1 inhibitors for treatment of HCC [116].

While STAT3 is phosphorylated in 60% of human HCC and active STAT3 correlates with tumor aggressiveness [118,119], no patients harboring STAT3 mutations have been described. However, activating STAT3 mutations have been described in iHCA patients [120]. These mutations are predominantly, but not exclusively, found in the SH2 domain of STAT3 and result in ligand-independent dimerization and Tyr705 phosphorylation of STAT3 and, consequently, in DNA binding and target gene expression [120,121]. Of note, mice that lack STAT3 expression in hepatocytes are protected from HCC [118]. Furthermore, the long noncoding RNA TSLNC8, which inhibits the phosphorylation of STAT3, is frequently downregulated in HCC patients. This RNA correlated with lower differentiation and patients with low expression of this RNA exhibited poorer survival [122].

In contrast to the tumor-promoting function of STAT3, there are indications that STAT1 might have rather tumor-suppressive effects [123,124]. In HCC patients, the majority of STAT1 is unphosphorylated, while phosphorylated STAT1 is responsible for the anti-tumor effect [125]. No STAT1 mutations have been described to correlate with HCC.

No mutations in the coding sequence of *SOCS3* are reported in HCC patients, but the promoter region is frequently hypermethylated, accompanied by reduced protein expression, which presumably contributes to the increase in STAT3 phosphorylation [126]. SOCS3 is downregulated specifically in HCC patients with a poor prognosis [119,127] and might be used as a marker to predict treatment response [128]. Expectedly, mice with a hepatocyte-specific SOCS3 deletion are more sensitive towards chemically induced HCC [129].

## 6. Therapeutic Opportunities for HCC Patients

Currently, several different treatment options exist for HCC patients (more detailed information can be found in recent reviews, e.g., [1,130,131,132]). An important therapy is the surgical resection of the tumor, although this is only indicated in patients in which hepatic function is preserved after partial hepatectomy and important co-morbidities of the patient like portal hypertension must be considered [21]. Other treatment options are local tumor ablation and transarterial therapies, in which transarterial chemoembolization (TACE) is the main method of choice [1]. TACE works by the infusion of chemotherapeutic agents coupled to embolic particles directly into an artery that is part of the tumors’ blood supply, thereby not only targeting the tumor cells by chemotherapy, but simultaneously reducing the oxygen provision of the HCC. A recent systematic review reported an objective response rate of 52.5% when HCC patients were treated with TACE [133]. Interestingly, there is also recent evidence that the serum levels of the cytokines IL-6 and IL-8 can be used as biomarkers to predict not only the tumor response, but also the overall survival after TACE [134].

The IL-6/Jak/STAT signaling axis offers multiple points for therapeutic intervention [135,136]. Best studied in this regard is IL-6 itself, which has been identified as a key therapeutic target in practically all inflammatory diseases [135]. Inhibitory antibodies against IL-6 or the IL-6R receptor are either already clinically approved or are in late-stage clinical development for different diseases, including rheumatoid arthritis, Castleman disease and cytokine release syndrome [135]. As mentioned above, sgp130Fc, which is also named Olamkicept and currently tested in phase II clinical trials in IBD patients, led to reduced tumor numbers in a mouse model of HCC and might, therefore, be a possible therapeutic opportunity [85]. However, mice with a hepatocyte-specific deletion gp130, the signal-transducing receptor of all cytokines of the IL-6 family, showed reduced progression, but no alteration in HCC initiation in the DEN model [107]. Whether targeting IL-6, IL-11 or one of the other family members has therapeutic value for human patients is currently unclear, but one might argue that inhibition of IL-6 might not be a suitable primary treatment option, but could be combined with one of the therapies outlined above. Nevertheless, a phase I/Ib study with AZD9150 (ISIS481464), an antisense oligonucleotide targeting STAT3, has been performed in 58 patients with advanced/metastatic HCC (ClinicalTrials.gov Identifier: NCT01839604). The drug appears to be tolerable, with serious adverse effects in four out of 15 patients that received the highest dose of 3 mg/kg body weight.

HCC patients can further be treated with systemic drugs. The multi kinase inhibitor sorafenib, which targets several kinases including c-Raf, VEGFR and PDGFR, was the first one that was approved by the FDA to treat HCC [1]. In a multicenter, phase 3, double-blind, placebo-controlled trial, the median overall survival was significantly higher for patients treated with sorafenib (10.7 months) compared to placebo (7.9 months) [137]. A number of other systemic drugs failed in clinical trials. However, most recently, lenvatinib, another multi kinase inhibitor that targets VEGFR1, 2 and 3, FGFR1, 2, 3 and 4, KIT, RET and PDGFRα, showed non-inferiority towards sorafenib in terms of median survival time (13.6 months vs. 12.3 months) [138]. Whether Janus kinase inhibitors, which are clinically approved for different inflammatory and immune diseases [139], could also be beneficial as a therapy in HCC is not known. At least in vitro, the Jak inhibitor ruxolitinib reduced proliferation and colony formation of HCC cells [140]. There is also anecdotal evidence from a variety of other small molecules that are presumably able to block the IL-6/Jak/STAT signaling axis, e.g., bazedoxifene, which is currently approved as a selective estrogen receptor modulator (SERM) [141]. It is currently unclear if and when such approaches will translate into the clinics.

## 7. Conclusions

Liver cancer, including HCC, is one of the leading causes of cancer-related deaths worldwide. Although progress has been achieved in the therapy and management of HCC patients, new additional approaches are warranted nevertheless.

Cytokines activating the Jak/STAT signaling cascade have been implicated in HCC initiation and development, and in particular IL-6 and IL-11 show encouraging results not only as potential biomarkers for liver tumors, but also as functional therapeutic targets. Initial studies demonstrating that blocking IL-6 or IL-11 in HCC reduces tumor burden in mice have recently been published [85,92], and these encouraging first results now must be confirmed and translated into the clinic. It is tempting to speculate that these cytokines also play important roles in other tumor entities of the liver. The same holds true for other proteins involved in IL-6 signaling like the metalloprotease ADAM17, which is not only recently identified and proposed as a target in lung cancer [142,143], but has also been implicated in HCC, where higher ADAM17 expression and the resulting activation of the receptor Notch1 correlated with poor prognosis of the patients [144].

In summary, targeting cytokines and, in particular, the Jak/STAT signaling cascade might be a useful therapeutic option for patients suffering from HCC.
